# Abnormally large malignant breast tumor with breast abscess: a rare clinical image

**DOI:** 10.11604/pamj.2022.42.315.36326

**Published:** 2022-08-26

**Authors:** Shrushti Yogeshwar Gawande, Jaya Gawai

**Affiliations:** 1 Department of Mental Health Nursing, Smt. Radhikabai Meghe Memorial College of Nursing, Datta Meghe Institute of Medical Sciences, Sawangi, Wardha, Maharashtra, India

**Keywords:** Breast cancer, pus, tumor, pain

## Image in medicine

Breast cancer develops from breast tissue. Signs of breast cancer may include a lump in the breast, a change in breast shape, dimpling of the skin, fluid coming from the nipple, a newly inverted nipple, or a red or scaly patch of skin. A 36-year-old female without any significant history came with the complaint of the sudden growth of breast tissues without pain in the last one month followed by pus discharge for one week along with red patches around the nipple. On physical examination, the tumor measured about 18cmx14cm. After investigations and biopsy, the patient was diagnosed with a malignant breast tumor.

**Figure 1:**
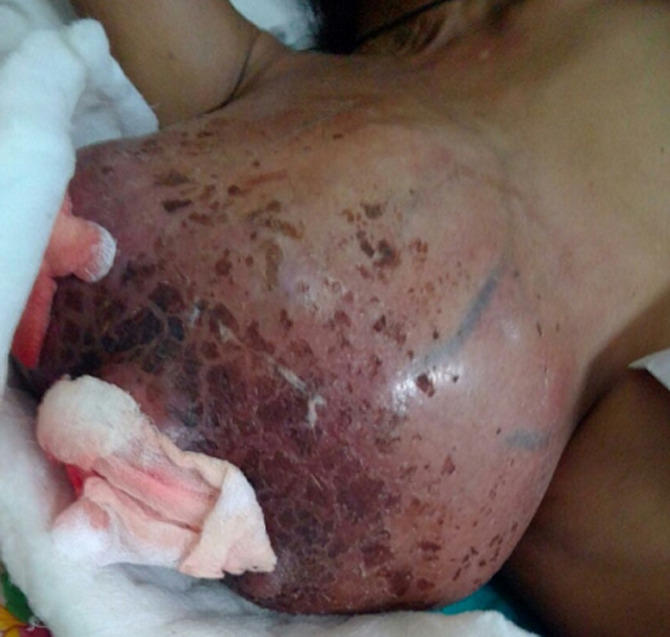
malignant breast tumor with breast abscess

